# Development, Implementation and Evaluation of an Educational Intervention to Prevent Low Speed Vehicle Run-Over Events: Lessons Learned

**DOI:** 10.3390/ijerph15040685

**Published:** 2018-04-05

**Authors:** Bronwyn Griffin, Kerrianne Watt, Roy Kimble, Linda Shields

**Affiliations:** 1 Centre for Children’s Burns and Trauma Research, Lady Cilento Children’s Hospital, South Brisbane, QLD 4101, Australia; royk@uq.edu.au; 2 School of Nursing, Queensland University of Technology, Brisbane, QLD 4059, Australia; 3 School of Public Health, Tropical Medicine and Rehabilitation Sciences, James Cook University, Townsville, QLD 4810, Australia; kerrianne.watt@jcu.edu.au; 4 Faculty of Medicine, University of Queensland, Australia, Herston, QLD 4006, Australia; 5 School of Rural Health, Charles Sturt University, Bathurst, NSW 2795, Australia; lshields@csu.edu.au

**Keywords:** low speed vehicle runover (LSVRO), child, adolescent, educational intervention, injury prevention and control, transport

## Abstract

There is a growing body of literature regarding low speed vehicle runover (LSVRO) events among children. To date, no literature exists on evaluation of interventions to address this serious childhood injury. Knowledge, attitudes, and behaviour regarding LSVROs were assessed via survey at a shopping centre (pre-intervention), then five months later (post-intervention), to investigate the effect of a population level educational intervention in Queensland, Australia. Participants’ knowledge regarding frequency of LSVRO events was poor. No participant demonstrated ‘adequate behaviour’ in relation to four safe driveway behaviours pre-intervention; this increased at post-intervention (*p* < 0.05). Most of the sample perceived other’s driveway behaviour as inadequate, and this reduced significantly (<0.05). Perceived effectiveness of LSVRO prevention strategies increased from pre- to post-intervention, but not significantly. TV was the greatest source of knowledge regarding LSVROs pre- and post-intervention. This study provides some evidence that the educational campaign and opportunistic media engagement were successful in increasing awareness and improving behaviour regarding LSVROs. While there are several limitations to this study, our experience reflects the ‘real-world’ challenges associated with implementing prevention strategies. We suggest a multi-faceted approach involving media (including social media), legislative changes, subsidies (for reversing cameras), and education to prevent LSVROs.

## 1. Background

The body and quality of literature involving unintentional childhood injury prevention interventions has developed substantially over the last 35 years. Persistence into research regarding the epidemiology and prevention of injuries incurred through drowning, hot water scalds, (bicycle) crashes (head injuries), and road traffic crashes (motor vehicle passenger and pedestrian), have resulted in significant culture changes and mandating of effective prevention strategies such as pool fencing legislation, hot water regulators, bicycle helmet use, car seat restraints, and traffic calming devices [1,2].

Yet there is room for improvement. Preventing low speed vehicle run over events (LSVRO) is one such challenge. The term LSVRO is used to describe incidents where a pedestrian—usually a child—is injured or killed by a slow moving vehicle (<30 km/h) in either a traffic or non-traffic area [3]. The setting of LSVROs is markedly different from higher speed pedestrian incidents which typically occur on public roads, therefore these events require focused prevention initiatives. There is a growing body of international evidence [4,5,6,7,8,9,10,11,12,13,14] in which the increasing burden of injury from LSVRO events, particularly among young children, is outlined. Published estimates of incidence of non-fatal LSVRO events range from 7–14.79 [7,15,16,17,18] per 100,000 per annum, and from 0.27–3.2 per 100,000 per annum for fatal events [11,13]. Trends over time were analysed in a recent comprehensive, population-based, retrospective cohort study of LSVROs conducted in Queensland, Australia [13]. Incidence of fatal and non-fatal (combined) LSVRO events among children aged 0–15 years increased over the study period (1999–2009). Importantly, no significant changes in mortality were observed for male or female (respectively) children aged 0–4, 5–9, or 10–15 years, during the 11 years of the study period. However, admission to hospital following an LSVRO event decreased for boys and girls aged 0–4 and 5–9 years, and increased for older children aged 10–15 years. Non-fatal incidents that did not result in hospital admission increased during the study period for boys and girls in each of the age groups. 

Literature suggests that drivers of vehicles involved in LSVROs are usually parents of the injured children [10,14,18,19,20,21,22]. While larger vehicles such as four-wheel-drives and sports utility vehicles are more frequently involved in fatal LSVROs, cars have been identified as the vehicle type most frequently involved in non-fatal events [14]. The location of these events is most frequently the home/driveway environment, but a substantial proportion occur on the street/public road. 

For all of these reasons, an educational intervention to increase knowledge, behaviours, and attitudes regarding LSVRO events is appropriate. Hence, the purpose of this study was to develop, implement, and evaluate an educational intervention to increase knowledge, attitudes, and behaviour of caregivers regarding prevention of LSVROs. 

### 1.1. Development of Intervention 

Initially, a community-based pre-post trial of two different communities (intervention vs. control), was planned to evaluate the impact of an educational campaign that would be developed based on the results of data analyses from an 11 year, retrospective, population-based, cohort study of the incidence and characteristics of fatal and non-fatal LSVRO events. It was planned to recruit participants via early childcare centres that provide care for 0–4 years old children within each community. Evaluation of the effect of the intervention was planned through investigating changes in parental attitudes, knowledge, behaviours, and practice. Focus groups were also planned to identify barriers to change and the acceptability of the intervention.

Due to unforeseen circumstances, the planned delivery and evaluation of the intervention changed course. In July 2010, a local public figure, Australian rugby union representative Brendan Cannon, ran over his son on the family property. The consequent attention by the media was very strong and prolifically portrayed. As the media attention on this topic was so widespread, it was clear that any chance of the planned community-trial would not be possible without substantial bias. Upon consultation and collaboration within the project group, injury prevention experts, Kidsafe QLD, and with the support of the QLD Government, it was decided to use the high profile incident as an opportunity to provide a public educational campaign regarding LSVRO prevention strategies. This was done by actively engaging with various levels of media including radio, newspapers, television (news and current affairs), internet news sites, blogs and social-media internet sites (note: our prevention message was mentioned in over 60 separate media reports during this time). Preliminary analyses of the aforementioned study provided sufficient evidence to design an educational campaign for the public to increase awareness of risk factors of LSVROs. 

### 1.2. Intervention

Effective supervision in child injury prevention requires a dynamic approach [23,24], including three major components (attention, proximity, and continuity). The intervention was based on this premise, together with the findings of a systematic literature review [11], and preliminary findings of the already described study. The intervention was designed to reflect the injury prevention model of Haddon’s Matrix, hence it focused on:

1. Host: small children (those most at-risk) are unaware of vehicle/road safety, not able to increase awareness until out of the at-risk age group, therefore focus must shift to raising awareness among caregivers.
2. Vehicle: all vehicle types must be considered, and visibility out of the car should be considered (including blind zones).
3. Physical Environment: Driveway design should incorporate the separation of a barrier to the driveway similar to pool separation.

**Figure A1:**
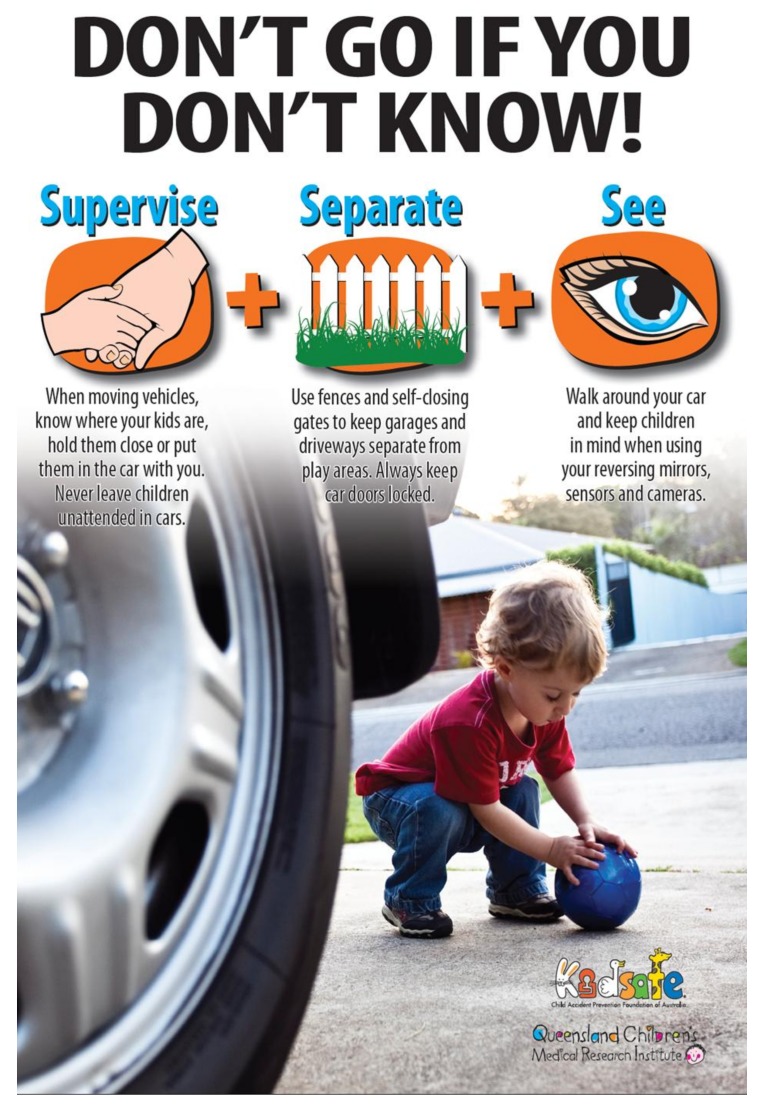
Poster.

**Figure A2:**
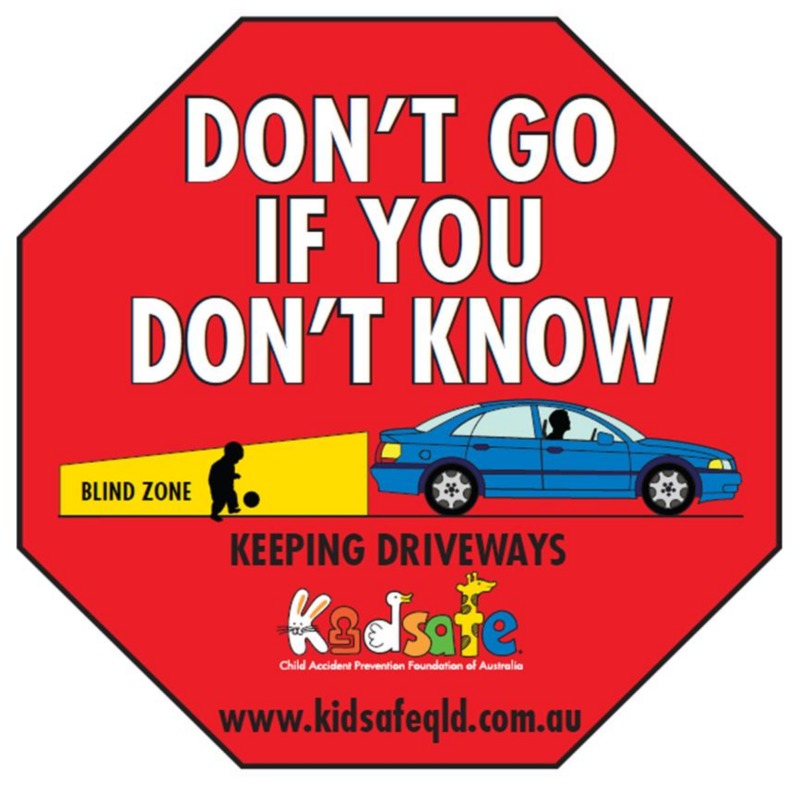
Sticker.

A poster and sticker were designed incorporating an engaging picture with a clear message of prevention (see Appendix A Figure A1 and Figure A2). The materials were prepared in collaboration with local injury prevention experts, and Kidsafe Queensland, with the input of a public relations expert and graphic design specialists. The aim was to prepare a clear, concise, evidence-based message that was accessible to the general public, but specifically care-givers of children most at-risk (0–4 years). 

The poster was sent to all early childhood education centres, kindergartens, and maternal child health units in the state of Queensland, as well as Kidsafe House (Queensland). Once the posters were visible in the community, they were requested and sent out to Queensland Police stations, Queensland Ambulance Service, and were also displayed throughout the Royal Children’s Hospital and the Mater Children’s Hospital in Brisbane (the two major hospital facilities for children in Queensland), and GP clinics throughout Brisbane. Kidsafe Queensland coordinated the poster printing and distribution.

## 2. Method

In order to assess population level changes in knowledge, attitudes, and behaviour regarding LSVROs due to the educational campaign, a pre-post design was used. A cross-sectional sample of the population was interviewed via face-to-face survey before posters were disseminated (pre-intervention) at a major shopping centre located near the Brisbane International Airport. The pre-survey was conducted over five day period (weekdays only) between 10 a.m.–3 p.m. The post-survey was conducted five months later at the same location, in the same manner. Pre- and post-surveys were conducted during non-school holiday periods; interviews were approximately 5–10 min. 

Participants were required to be aged over 16 years, hold a driver’s license, be a resident of Queensland, and provide informed consent.

Trained interviewers (wearing brightly coloured t-shirts provided by Kidsafe) were stationed in the Food Court (which is located in the centre of the shopping centre, directly adjacent to an entry/exit to the centre), and approached passing shoppers. Interviewers attempted to speak to every passing shopper potentially eligible to participate in the study. Interviewers introduced themselves, provided a brief explanation of the study, requested consent to participate, and proceeded with the interview if consent was provided. If consent was not provided, the next available shopper was approached. Upon completion of the survey, all participants were thanked for their involvement and advised of the number of LSVRO incidents among children in Queensland per week, the age-group most at-risk, and the suggested strategies for preventing LSVRO incidents. 

### 2.1. Survey Instrument 

A copy of the two-page, 17 item survey instrument can be obtained from the authors on request. Data collected in the survey included:

- Demographic characteristics: Participants provided information on gender, age, post-code (later converted into a dichotomous measure of accessibility to major cities/services), education level, income, relationship status, main language spoken at home, ethnicity, and employment status. Participants were also asked about their caregiving responsibilities for children. Participants were asked whether they lived with children, whether they cared for children in their own (participants’) home, whether they cared for children in the children’s home, and the ages of these children.
- LSVRO environmental risk factors: Driveway features (whether driveway is separated from children’s access; whether driveway is used as a play area; and whether driveway is used for reversing vehicles) were assessed via a five-point Likert scale (1 = never; 5 = always), which were then dichotomised (never vs. any other response) for each item.
- LSVRO Knowledge/attitudes/behavior: Knowledge of frequency of LSVRO events and the age group most at risk were assessed via multiple choice questions, which were each categorised into correct or incorrect. *Driveway* behaviour was measured using a five-point Likert Scale (1 = never; 5 = always)—participants rated how frequently they performed a list of four safety behaviours (walk around car, look around car, lock car, and physically hold the child when someone else is reversing) before reversing their vehicle in the presence of children. Responses of 1–3 were allocated a score of 0, and other responses a score of 1; scores were then summed (≥3: adequate behaviour, <3: inadequate behaviour). Originally use of a reversing mirror was included in the list of behaviours but this was not used in the calculation of total score for driveway behaviour, because the majority of the sample did not have a reversing camera on their vehicles.
- Perceived behaviour of others: Participants rated how frequently they perceived that others perform the same list of four behaviours before reversing their vehicle in the presence of children; the same approach was used to calculate a total score. The item regarding use of a reversing camera was analysed as a separate variable (infrequent: never, sometimes, about half the time, frequently, usually, or always).
- Perceived Effectiveness: Participants rated the same five behaviours in terms of their perceived effectiveness in preventing an LSVRO incident (1—not at all effective; 5–100% effective). The mean scale score was calculated and categorised: <3.5: “low perceived effectiveness”; ≥3.5: high perceived effectiveness. Reliability analyses confirmed internal reliability of this measure (Chronbach’s alpha = 0.8). In addition, participants were asked two open-ended questions:
  ○ Are there any strategies you use or think might be effective to prevent driveway run-overs?
  ○ What reasons do you think people might give for not using safe strategies in the driveway?
    Responses to these questions were recorded verbatim and later grouped into eight themes.
- Intervention Awareness: Participants were asked to nominate from a list of sources whether they had seen or heard about driveway run-overs in children. To allow analyses of each of these potential sources of awareness regarding LSVROs, 10 separate variables were created (yes/no for each). In addition, one overall variable was created to describe whether participants had heard of LSVRO from any source (yes/no).

### 2.2. Statistical Analysis 

Descriptive analyses were used to describe the overall characteristics of the sample, and to ascertain whether there were any differences in demographic characteristics between the pre- and post-intervention sample. Changes from pre-intervention to post-intervention on the five measures of LSVRO-related knowledge, attitudes, and behaviour were assessed using nonparametric tests (chi-square). Changes in awareness from pre- to post-intervention regarding the LSVRO intervention were also assessed by chi-square analyses, or in cases where assumptions were violated a Fisher’s exact test was used. Data were analysed using SPSS Version 21 (IBM, Armonk, NY, USA) [25]. 

## 3. Results

### 3.1. Sample Characteristics

The pre-intervention sample comprised 135 participants, and the post-intervention sample comprised 206 participants. Sample characteristics are summarised in Table 1. 

The sample differed between pre and post-intervention on several demographic characteristics. There were significantly more 26–34 years old in the post-survey (n = 91; 44%) compared with the pre-survey (n = 37; 28%; *p <* 0.05). Significantly more participants in the post-survey were in a permanent relationship (*p* < 0.05), and significantly fewer reported regularly looking after children in their own home, or in the childrens’ homes (*p* < 0.05).

### 3.2. LSVRO Knowledge, Attitudes, and Behaviour 

Knowledge, attitudes, and behaviour of the sample relating to LSVRO incidents are reported in Table 2. Overall, knowledge regarding frequency of LSVRO incidents in Queensland was poor. Over three-quarters (76.9%; n = 126) of the sample provided an incorrect response to this item pre-intervention, and 61.2% of the post-intervention sample provided an incorrect response (n = 126; this difference was significant X^2^ = 9.10; df = 1; *p* < 0.01). In contrast, three-quarters of the sample were able to correctly identify the age-group most at risk for LSVRO incidents pre-intervention (n = 99), and this did not change significantly between pre- and post-intervention (*p* > 0.05). 

At baseline, not one respondent was categorised into ‘adequate behaviour’ in relation to the four safe driveway behaviours. The proportion of respondents categorised into ‘adequate behaviour’ increased substantially at post-intervention (n = 102; 50.7%) (no statistic is provided due to one cell size being 0). 

At base-line, 18.4% of participants rated other’s driveway behaviour as adequate, based on the four safe measures of driveway behaviour (n = 23), and this reduced significantly at the post-intervention survey (10.6%; n = 21; X^2^ = 3.84; df = 1; *p* = < 0.05). 

The perceived effectiveness of LSVRO prevention strategies increased from pre- (n = 78; 59.1%) to post-survey (n = 139; 68.8%), however this was not a significant increase (*p* > 0.05). 

When asked how frequently others use a reversing camera when children are around, at base line the majority of respondents indicated that others use reversing cameras infrequently (94.4%; n = 119). This decreased slightly at post-intervention (87.9%, n = 174). The association bordered on significance (*p* = 0.05). 

### 3.3. LSVRO Environmental Risk Factors 

Changes in LSVRO environmental characteristics were also evaluated to determine whether there was any effect of the intervention (Table 3). The proportion of respondents who reported that their driveway was never separate from children’s access decreased slightly from pre-intervention (59.7%) to post-intervention (51.8%), but this was not a significant difference (*p* > 0.05). Similarly, the proportion of respondents who reported that their driveway was never used as a children’s play area increased from pre-intervention (68.2%) to post-intervention (74.8%), but again this was not significant (*p* > 0.05). The majority of respondents indicated that their driveway was used to reverse vehicles in/out of, and this was not significantly different between pre- (96.9%) and post-intervention (96.6%). Overall, 10.6% (n = 36) of the sample reported having a reversing camera in their car (this did not differ at pre- or post-intervention; *p* > 0.05). 

### 3.4. Reasons for Unsafe Driveway Behaviour 

**Figure 1:**
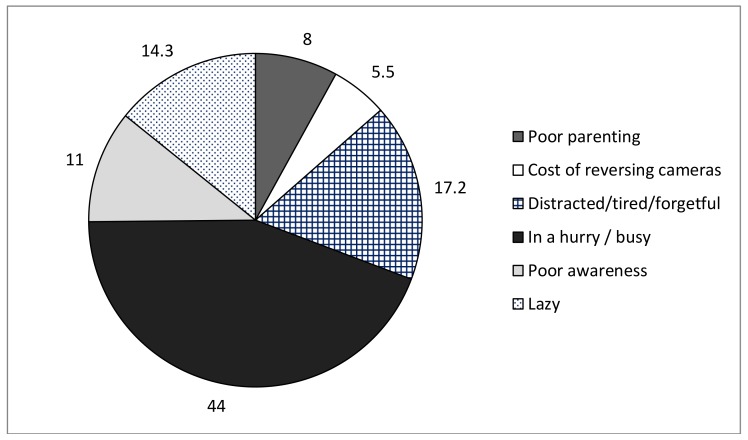
Reasons that others do not use safe strategies in the driveway (%).

Reasons provided by participants for other drivers not using safe strategies in the driveway are shown in Figure 1; the primary reason provided was ‘being too busy or in a rush’ (44%), followed by ‘being tired, distracted, or forgetful’ (17.2%). 

### 3.5. Strategies for Preventing LSVROs 

**Figure 2:**
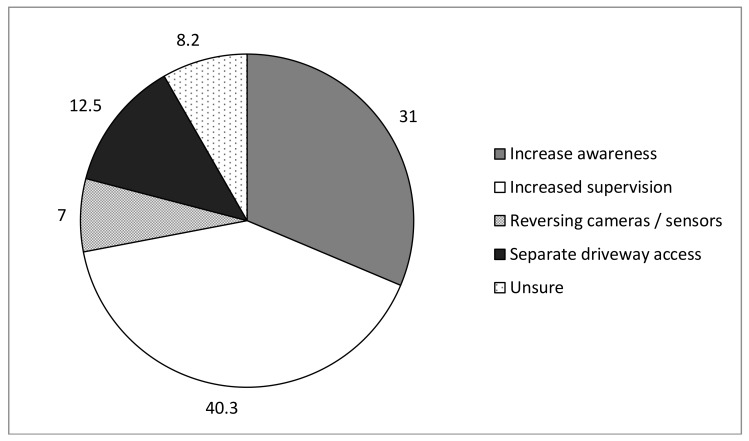
Strategies suggested by respondents for reducing LSVRO incidents (%).

**Figure 3:**
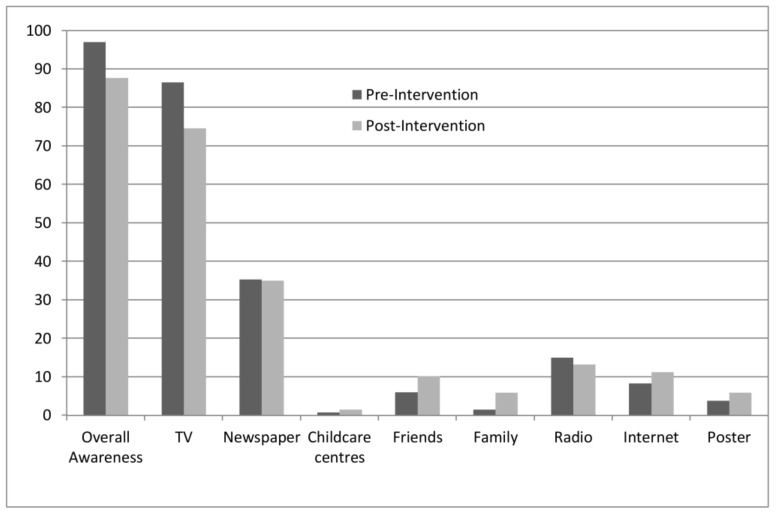
LSVRO Awareness at pre-intervention and post-intervention.

Suggestions for strategies to reduce LSVRO incidents are shown in Figure 2. Forty-one percent of the sample suggested that increasing supervision of children would be an effective strategy. This was followed by increasing awareness about LSVRO incidents (31.6%) (note: advertising and education were the most frequently suggested mechanisms). Interestingly, the proportion of respondents who suggested that separating the driveway from areas accessible to children increased from 5% at pre-intervention (n = 5) to 20.7% at post-intervention (n = 23). 

(Numbers shown in Figure 2 and Figure 3 relate to all survey participants from pre- and post-intervention). 

### 3.6. Awareness of Intervention 

Figure 3 shows that the most common source of information regarding LSVRO incidents among survey respondents, was the television (86.5% at baseline). The next most common source of information was the newspaper (35.3%). 

Between pre-intervention and post-intervention, overall LSVRO awareness among survey respondents decreased (pre: 97.0% to post: 87.7%; X^2^ = 9.04; df = 1; *p* < 0.01). A decrease was also observed in the proportion of respondents who had seen or heard about LSVROs through TV (pre: 86.5% vs. post: 74.6%; X^2^ = 6.88; df = 1; *p* < 0.01). Some increases were observed between pre- and post-intervention in relation to LSVRO awareness. Compared with pre-intervention, more respondents reported having seen or heard about LSVROs at childcare centres; through family or friends, and via the internet. However, these differences were small and not significant (Figure 1). At pre-intervention (before the poster was designed and released), 5 (3.8%) respondents reported that they had seen the poster, and at post-intervention, 12 respondents (5.9%) reported that they had seen the poster. 

## 4. Discussion 

This study was designed to evaluate the impact of a population-level educational intervention to increase awareness regarding LSVRO incidents. This is the first survey known to the authors that has assessed knowledge, attitudes, and behaviour in relation to LSVRO incidents. 

Overall, participants in the study demonstrated poor knowledge regarding the frequency of LSVRO incidents, but knowledge of the age-group most at risk was high. Alarmingly, at baseline, not one respondent was categorised into ‘adequate behaviour’ in relation to the four safe driveway behaviours (walk around car, look around car, lock car, physically hold the child when someone else is reversing). Approximately one in five participants provided responses that categorised others’ driveway behaviour as adequate, using the same four measures. When asked to rate the effectiveness of these four strategies (with the addition of using a reversing camera) in relation to reducing LSVRO incidents, the majority of participants perceived that the strategies were effective (59.1%). Nearly all respondents indicated that their driveway was used to reverse vehicles in/out of (96.6%). 

The findings of this study provide some evidence that the educational campaign and opportunistic media engagement was successful in increasing awareness and improving behaviour regarding LSVRO incidents. Knowledge of LSVRO frequency increased significantly from pre- to post-intervention, and the proportion of respondents whose driveway behaviour was categorised as adequate increased from none at baseline to fifty-one percent at post-intervention. It is noted that this finding is extreme, and we acknowledge that it is possible that some form of interviewer bias may have impacted on this finding. Interviewers were trained in survey administration and briefed prior to the data collection, however the possibility of bias cannot be ruled out. Interestingly, significantly fewer participants rated others’ driveway behaviours as adequate at post-intervention, compared with pre-intervention. This may reflect increased awareness and understanding of the four safe driveway behaviours in the general population. The perceived effectiveness of LSVRO prevention strategies increased from pre to post-survey. Although the increase was not significant, it is nonetheless encouraging. 

Only 10% of the sample reported having a reversing camera in their car. Nearly all respondents indicated that their driveway was used to reverse vehicles in/out of (96.6%). Over half of the sample reported that their driveway was never separate from children’s access (59.7%), and this did not change at follow-up. More respondents reported that their driveway was never used as a children’s play area at post-intervention than pre-intervention, but this was not a significant increase. 

The awareness of LSVRO incidents increased at a population level from pre- to post-intervention however strategies to prevent them did not. Given this gap in knowledge of strategies, it is plausible that the increased knowledge of LSVRO incidents was an effect of increased media attention during the period rather that exposure to the poster. It is evident that there is much more work to be done to target effective, sustainable prevention strategies. 

The increased awareness can not necessarily be attributed to the educational intervention (poster). The survey results indicate that fewer participants had seen or heard of LSVROs at post-intervention than at baseline. This reduction in awareness can mainly be attributed to the reduced media attention (specifically television, and to a lesser extent radio). The media attention was at its peak in the months immediately prior to the pre-survey. Thus, it is not surprising that fewer participants reported seeing or hearing about LSVRO incidents via the media at the follow-up survey five months later. However, some increases were observed between pre- and post-intervention in relation to LSVRO awareness. While the numbers were too small to warrant analyses, more respondents reported having seen or heard about LSVROs at childcare centres; through family or friends, and via the internet at the post-intervention survey than at pre-intervention. Of most relevance is that more respondents reported seeing the poster at post-intervention, than at pre-intervention (3.8% vs. 5.9%). Five respondents indicated that they had seen the poster at baseline (before it had been distributed). It is possible that these respondents had seen a different LSVRO poster than the poster designed for the educational intervention under examination in the present study. It is also possible that some participants misunderstood this question because English was not their primary language (this was suggested by survey interviewers). 

The study design used to evaluate this intervention is not ideal. Different populations were surveyed pre-intervention and post-intervention, and there was no control group. Thus, it is not possible to determine whether the observed changes in LSVRO knowledge, attitudes, and behaviour are attributable to the educational intervention. The original study design (pre-post community-trial, where the same population would have been surveyed before and after intervention implementation, and included a control group), would have provided stronger evidence regarding the effectiveness of the intervention. However, for the reasons outlined at the beginning of this section, it was not possible to proceed with the original design. This was the only option possible in the very short timeframe available to opportunistically build on the intense media attention on the LSVRO issue at the time. 

Although there was intense media attention and great demand/interest/pressure from many sources to produce and distribute the posters, the time of year was not preferable for such an intervention. Posters were distributed in December. This is towards the end of the year, which is busy for many reasons. Even though the posters were distributed to all of the locations described earlier, there is no way of knowing whether the posters were actually put up at the locations where the posters were sent. Childcare centres were one of the main distribution points, and many children do not attend child care, or attend in a lesser capacity, toward the end of the year. In addition, in January 2011, south-east Queensland experienced a major flood event. Thus, there were several significant independent events that occurred during the period of intervention implementation that may have minimised the impact of the intervention. 

### 4.1. Effective Strategies to Increase Awareness 

The findings of this study suggest that it is beneficial to incorporate television media, and to a lesser extent, newspaper media, to increase population level awareness regarding LSVRO incidents. The main source of information reported by participants regarding LSVRO incidents was the television (86%), followed by the newspaper (35%). The use of high profile members of society and real life events may also increase population awareness. Anecdotally, an outstanding majority of participants referred to high profile Queensland athletes who had been on TV and newspapers because of LSVRO incidents involving their young children. Participants commonly remembered these stories in both surveys, even on the post-intervention survey, which was almost one year after the last incident. Based on these findings, a Community Service Announcement was later created via Kidsafe Queensland, which included Brendon Cannon, as well as the Brisbane Broncos (a high profile Queensland football team). However such exercises are expensive and rely on (1) goodwill of organisations to provide sponsorship to cover production costs, and more prohibitively (2) television stations for broadcasting. 

Education alone is insufficient to effect behaviour change. Klassen [1] argues that education should occur in the context of improved skill, changes in social norms and a supportive environment, and reinforcement that encourages behaviour change. Relatedly, Hanson suggests that physical, psychological and sociological dimensions of injury must be addressed at the community level to reduce injury, Hanson suggests that physical, psychological, and sociological dimensions of injury must be addressed at the community level to reduce injury [26]. Morris and Trimble found that a combination of education and a subsidy (towards helmet purchase) significantly increased implementation of an intervention designed to increase helmet wearing when riding a bicycle [27]. Hunter and colleagues (2014) also demonstrated that the an integrated educational program, together with subsidised restraints, is effective in improving uptake of best practice behaviours with respect to child restraints, particularly among communities with a high proportion of low-income families [28]. Most recently Burgess et al. described their experience using social media recruitment methods used to enroll mothers of young children to an app-based burn prevention intervention. They found this methodology to be rapid and cost-effective recruitment of participants with social, geographic, and economic diversity that were largely representative of the population [29,30]. Therefore, we hypothesise that a multi-faceted approach involving media (including social media), legislative changes, subsidies (for reversing cameras), and education are required to prevent LSVROs (and injuries overall). 

### 4.2. Limitations

Aside from those already discussed, this study had limitations. Differences were observed on several demographic characteristics in the baseline sample and post-intervention sample. Thus, attributing any observed differences in outcome measures to the intervention should be done with caution. It is possible to investigate this further by conducting stratified analyses (e.g., differences in the outcome measures at pre- and post-intervention by age group).

Measurement bias may also be present in this study. Information was gathered using a structured interview, thus interviewer bias is possible. Recall bias is possible for those items requiring respondents to rely on their memories of events. It is likely that selection bias was present in this study, due to the location and time period of data collection. Participants were interviewed at a shopping centre during the weekdays, between 10 a.m.–5 p.m. Although interviewers were briefed in relation to recruitment strategies, volunteer bias is also possible. Comparison of the sample characteristics with the characteristics of the general Queensland population revealed some differences. Overall, in comparison to the general population of Queensland, our sample comprised more females, more people who speak English as the primary language at home, more people with no children; and slightly less people employed full-time. Also caution should be used when drawing comparisons between pre and post intervention as they were two different groups.

Most of these biases would have been avoided had the original study design been used. However, under the circumstances, it was not possible to conduct the originally planned evaluation. We instead embraced the public educational campaign afforded us by the rare opportunistic media engagement resulting from the generosity of one family who experienced the tragedy of a LSVRO, in order to increase awareness of this event. We proceeded with the evaluation described herewith, rather than not conducting any evaluation at all. 

## 5. Conclusions 

This study evaluated the impact of an educational intervention on knowledge, attitudes, and behaviour related to LSVROs. It is the first study known to the authors where knowledge, attitudes, and behaviour of the public regarding LSVRO incidents have been investigated. While there are several limitations to this study, some differences were observed between pre-intervention and post-intervention that suggest increased awareness at a population level regarding LSVRO incidents and strategies for prevention. Multiple strategies are required to prevent incidence and injury from LSVROs. 

## Figures and Tables

**Table 1:** Population-Based Survey Sample Characteristics.

Demographic Characteristics		Pre-Intervention n = 135	Post-Intervention n = 206
		n (%)	n (%)
Gender X^2^ = 1.29; df = 1; *p* > 0.05	Male Female	53 (39.3%) 81 (60.4%)	69 (33.5%) 137 (66.5%)
Age * (n = 3 missing) X^2^ = 11.29; df = 3; *p* < 0.01)	16–25 years 26–35 years 36–45 years 46+ years	35 (23.6%) 37 (27.8%) 33 (24.8%) 28 (21.1%)	32 (15.6%) 91 (44.4%) 42 (20.5%) 40 (19.5%)
Remoteness ^a^ (n = 21 missing) X^2^ = 4.97; df = 1; *p* > 0.05	Highly accessible Not highly accessible	114 (95%) 6 (5%)	189 (94.5%) 11 (5.5%)
Relationship status * (n = 8 missing) X^2^ = 35.92; df = 1; *p* < 0.001	Living with a partner Not living with a partner	45 (34.4%) 86 (65.6%)	137 (67.8%) 65 (32.2%)
Education (n = 13 missing) X^2 =^ 3.47; df = 3; *p* > 0.05	≤Grade 10 Grade 12 TAFE University	22 (16.4%) 35 (26.1%) 27 (20.1%) 50 (37.3%)	21 (10.8%) 49 (25.3%) 35 (18.0%) 89 (45.9%)
Main language at home (n = 3 missing) X^2^ = 1.78; df = 1; *p* > 0.05)	English Other	120 (90.9%) 12 (9.1%)	195 (94.7%) 11 (5.3%)
Household income (Gross, per annum) (n = 38 missing) X^2^ = 1.76; df = 3; *p* > 0.05)	<$26,000 $26,000–67,499 $67,500–114,999 $115,000+	14 (11.2%) 44 (35.2%) 39 (31.2%) 28 (22.4%)	18 (10.1%) 52 (29.2%) 59 (33.2%) 49 (27.5%)
Employment (n = 16 missing) X^2^ = 1.03; df = 2; *p* > 0.05)	Full-time Part-time/Casual Not employed	69 (51.5%) 27 (20.1%) 38 (28.4%)	98 (50.8%) 32 (16.6%) 61 (32.6%)
Number of children live with X^2^ = 0.76; df = 3; *p* > 0.05	None 1 2 3+	74 (54.8%) 19 (14.1%) 30 (22.2%) 12 (8.9%)	119 (57.8%) 27 (13.1%) 39 (18.9%) 21 (10.2%)
Care for children in OWN home (monthly or more) * (n = 5 missing); X^2^ = 4.62; df = 1; *p* < 0.05	Yes No	66 (50.8%) 64 (49.2%)	80 (38.8%) 126 (61.2%)
Care for children in THEIR home monthly or more * (n = 4 missing); X^2^ = 17.64; df = 1; *p <* 0.001	Yes No	68 (51.9%) 63 (48.1%)	60 (29.1%) 146 (70.9%)

^a^ Geographical remoteness was measured through ARIA (Accessibility/Remoteness Index of Australia). Each geographical area (defined by postcode) was allocated a score between 0 and 15, based on the (road) distance to nearby towns that provide services. Scores were then allocated to the following categories (OESR Queensland, 2011): Major city: 0.0–0.2; Inner Regional: 0.2–2.4; Outer Regional: 2.4–5.92, Remote: 5.92–10.53; Very Remote: 10.53+). * indicates significant difference between pre- and post-intervention.

**Table 2:** LSVRO Knowledge, Attitudes, and Behaviour.

Outcome Variable		Pre-Intervention n = 135		Post-Intervention n = 206	
		n	%	n	%
Knowledge					
LSVRO Frequency * (n = 1 missing; X^2^ = 9.10; df = 1; *p* < 0.01)	Low	103	(76.9%)	126	(61.2%)
	High	31	(23.1%)	80	(38.8%)
**Age group most at risk** (n = 4 missing; X^2^ = 0.02; df = 1; *p* > 0.05)	Low	33	(25%)	53	(25.7%)
	High	99	(75%)	153	(74.3%)
Behaviour					
Driveway Behaviour * (n = 11 missing)	Inadequate	129	(100%)	99	(49.3%)
	Adequate	0	(0%)	102	(50.7%)
Perceived behaviour of others * (n = 11 missing; X^2^ = 3.96; df = 1; *p* < 0.05)	Inadequate	102	(81.6%)	177	(89.4%)
	Adequate	23	(18.4%)	21	(10.6%)
Attitudes					
Perceived others’ use of reversing camera * (n = 17; X^2^ = 3.84; df = 1; *p* = 0.05)	Infrequent Frequent	119 7	174 24	(94.4%) (5.6%)	(87.9%) (12.1%)
**Perceived effectiveness of LSVRO strategies** (n = 7 missing; X^2^ = 3.31; df = 1; *p* > 0.05)	Low	54	(40.9%)	63	(31.2%)
	High	78	(59.1%)	139	(68.8%)

* indicates significant difference between pre- and post-intervention.

**Table 3:** LSVRO Environmental Risk Factors.

LSVRO Environmental Risk Factor		Pre-Interventionn = 135	Post-Interventionn = 206
		n (%)	n (%)
Driveway—separate access ^a^ (n = 23 missing; X^2^ = 1.88; df = 1; *p* > 0.05)	Never Other	71 (59.7%) 48 (40.3%)	103 (51.8%) 96 (48.2%)
Driveway—play area ^b^ (n = 3 missing; X^2^ = 1.73; df = 1; *p* > 0.05)	Never Other	90 (68.2%) 42 (31.8%)	154 (74.8%) 52 (25.2%)
Driveway—reversing ^c^ (n = 9 missing; X^2^ = 0.03; df = 1; *p* > 0.05)	Never Other	4 (3.1%) 125 (96.9%)	7(3.4%) 196 (96.6%)
Reversing camera in car	Yes No	17 (12.6%) 118 (87.4%)	19 (9.2%) 187 (90.8%)

^a^ Participants responded to the question “is your driveway separated from children’s access?” on a five-point likert scale (1 = never; 5 = always). Responses were dichotomised into: never; other. ^b^ Participants responded to the question “is your driveway used as a play area for children?” on a five-point likert scale (1 = never; 5 = always). Responses were dichotomised into: never; other. ^c^ Participants responded to the question “is your driveway ever used to reverse vehicles in/out of? on a five-point likert scale (1 = never; 5 = always). Responses were dichotomised into: never; other.
